# Interlayer Shear Characteristics of Bridge Deck Pavement through Experimental and Numerical Analysis

**DOI:** 10.3390/ma15197001

**Published:** 2022-10-09

**Authors:** Weidong Chen, Bing Hui, Ali Rahman

**Affiliations:** 1 Chang’an-Dublin International College of Transportation, Chang’an University, Xi’an 710064, China; 2 School of Highway, Chang’an University, Xi’an 710064, China; 3 School of Civil Engineering, Southwest Jiaotong University, Chengdu 610031, China; 4 Key Laboratory for Highway Engineering of Sichuan Province, Southwest Jiaotong University, Chengdu 610031, China

**Keywords:** bridge deck pavement, interlayer bond strength, adhesive material, emulsified asphalt, shear stress

## Abstract

In order to study the interlayer shear behavior of bridge deck pavement, a numerical simulation was conducted to analyze the influence of varying interfacial conditions on shear stress at the bottom of pavement layers under the moving loading effect. Moreover, the shear strength of the different adhesive and waterproof adhesive materials was evaluated by conducting laboratory tests. The results showed that improving the bonding condition at the upper interlayer led to the reduction of the shear stress at the bottom of the pavement layers. With the increase of the friction coefficient of the upper interface to the full bonding state, the resulting shear stress at the bottom of the upper layer declined to the lowest value, which was about 35% of that of the full slip state. When the lower interlayer was in the full slip or partial bonding state, the resulting shear stress at the bottom of the lower layer decreased linearly with the increase of the friction coefficient of the upper interlayer. Moreover, once the contact state of the upper interlayer reached the full bonding state, the resulting shear stress at the bottom of the lower layer reached the minimum, which is about 88% of that of the full slip state. To improve the integrity and shear resistance of the bridge deck pavement structure, interlayer bonding should be strengthened. In this regard, the resin emulsified asphalt was determined as an appropriate adhesive material to be applied at the upper and lower interlayers. In addition, interlayer shear bond strength, regardless of the type of adhesive materials, was decreased with increasing temperature. Finally, statistical analysis results indicated that all factors of structure type, type of adhesive material, and temperature statistically have a significant effect on interlayer bond strength. The findings of this study could provide a theoretical basis and experimental support for improving the interlayer design and construction in the concrete bridge deck pavement structure.

## 1. Introduction

Due to direct contact with traffic loading and environmental circumstances, the characteristics of the bridge deck pavement play an important role in the performance of the bridge structure. In recent years, many studies have been conducted to improve the performance and functionality of bridge deck pavement. The type and properties of materials used in the bridge deck pavement are one of the key factors in the overall performance of the bridge deck pavement. Epoxy asphalt mixture is one of the most widely used pavement materials in the construction of many large-span steel bridges because of its excellent anti-deformation ability, fatigue resistance, water stability, and corrosion resistance. Many studies were conducted to improve the performance of this type of asphalt mixture in the bridge deck pavement. A novel study proposed an approach of using polyurethane (PU) and epoxy resin (EP) to prepare an epoxy/polyurethane (EPU) modified asphalt binder and mixture. The results showed that the low-temperature cracking resistance of EPU-modified asphalt mixture was significantly improved compared to epoxy asphalt mixture, exhibiting a good application prospect in flexible bridge deck pavement engineering [1]. Several methods such as optimizing the skeleton structure or developing a cold-mixed ultra-thin antiskid surface layer (UTASS) were also successfully devised to improve the skid resistance of the epoxy asphalt-based concrete (EAC) to be used as a surface layer for the bridge deck pavement [2,3].

To improve the low-temperature and fatigue performances of the bridge deck pavement and decrease its cost, Zhang et al. [4] developed an unsaturated polyester resin (UPR) modified asphalt mixture. Their experimental results showed that the laboratory performance of UPR modified asphalt mixture outperformed SBS modified asphalt mixture and epoxy-modified asphalt mixture. In addition, the cost of UPR-modified asphalt was only 62% of that of epoxy-modified asphalt. Other studies demonstrated that the incorporation of UPR-emulsion could solve the problems associated with durability reduction and service-life shortening of bridge deck pavement concrete [5]. The application of sustainable pavement materials to improve the longevity of orthotropic bridge deck pavement structures has been promising. For instance, characteristics of a newly developed thermosetting polyurethane modified asphalt binder (TPUA) revealed its excellent high-temperature deformation resistance and elastic recovery properties [6]. The problem of early damage to concrete bridge deck pavement in cold regions has become one of the major problems for bridges. To address this issue, Cheng and Shi [7] used nanotechnology material by the addition of Nano-SiO_2_ into the concrete to enhance its durability. The test results indicated that the incorporation of nano-SiO2 greatly improved the four durability indexes, strength, frost resistance, resistance to Cl^−^ ion permeability, and abrasion resistance of the modified concrete. In another attempt, a polyurethane concrete (PUC) consisting of a one-phase tough polyurethane binder as the matrix and dolerite aggregates as filler was successfully developed to be used in bridge deck pavement. The findings showed that PUC composites have strong low-temperature toughness, high dynamic stability, and above all excellent durability [8].

The evaluation of the mechanical performance of pavement bridge deck has been a matter of concern among researchers due to complex service conditions, such as composite structure design, massive vehicle loadings, environmental conditions, large vibration, and large deflection deformation. Wang et al. [9] conducted a numerical analysis using the finite element method (FEM) to study both the static and dynamic stress in bridge deck pavement. Their results showed that the suggested thickness for the upper layer pavement is 3.5 cm to 4.5 cm and the lower layer is 5 cm to 7 cm. Moreover, the suggested modulus for the pavement upper layer is 1600 to1900 MPa and 900 to 1000 MPa for the lower pavement layer. In recent years, the application of ground penetration radar (GPR) as a non-destructive testing (NDT) method has become a viable technique for proper monitoring and early diagnosis of distresses such as moisture damage in bridge deck pavement [10].

The quality of the bonding between pavement layers directly affects the integrity and performance of pavement structure. Previous studies [11,12,13,14,15,16] applied the FEM to analyze the mechanical responses of the pavement structure under different interlayer contact and loading conditions. The structure of a bridge deck pavement, as a typical layered structure, from top to bottom is generally composed of a surface layer, bonding layer, lower layer, waterproof bonding layer, and cement concrete bridge deck. It was found that, owing to the differences in material properties and defects during the construction of bridge deck pavement, the problem of insufficient bonding between layers is common, which will reduce the shear capacity of layers [17,18,19,20,21,22,23]. The bridge deck pavement is different from the road pavement in terms of stress state, environmental, and service conditions. Therefore, it is more prone to various distresses, and the performance of the pavement directly affects the durability of the bridge, safety, and comfort of driving [24,25]. The current research on the bonding performance of bridge deck pavement is mostly focused on the upper interlayer or the lower interlayer exclusively [19,26,27,28,29]. The factors affecting interlayer bonding performance considered in previous studies are mainly divided into internal factors (including type and dosage of bonding material [24,26,30], interface roughness [31]), and external factors (including ambient temperature, construction conditions, traffic load) [17,32,33]. Indoor tests and finite element analysis are commonly used as effective methods to study interlayer bonding performance [34]. Shear strength and tensile strength are often used as evaluation indicators [23]. The above-mentioned studies provide valuable experience in improving the overall quality of the interlayer bonding between pavement layers. However, these studies have mainly focused on the performance of bonding or waterproof bonding layers, and interlayer bonding behavior in the presence of two adjacent layers is not investigated thoroughly. In view of this research gap, this study aims at analyzing the influence of the adhesive layer and waterproof bonding layer on mechanical responses of the bridge deck pavement under dynamic loading through numerical simulation. Moreover, in order to improve the shear resistance between the layers of the pavement structure, the shear strength of different adhesive materials was studied through laboratory experiments, so as to provide experimental support for the rational material selection of interlayer treatment.

## 2. Numerical Simulation

The finite element method (FEM) is a popular numerical method for solving partial differential equations arising in engineering and mathematical modeling in two or three space variables. The FEM partitions a large system into smaller, simpler parts that are called finite elements to solve a problem. This is achieved by a particular space discretization in the space dimensions, which is implemented by the construction of a mesh of the object [35]. In this study, the FEM was utilized to study the influence of the adhesive layer and waterproof bonding layer of the concrete bridge deck pavement on the mechanical responses of the upper and lower layers of the pavement bridge deck.

### 2.1. FEM Model Development

#### 2.1.1. Model Structure

**Figure 1:**
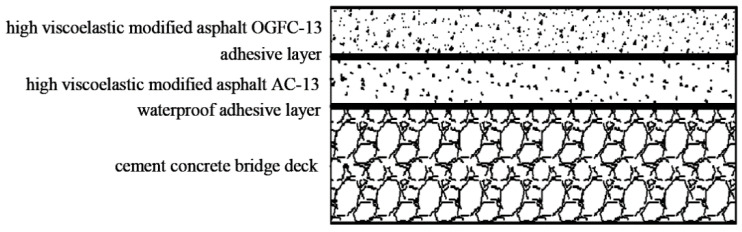
Bridge deck pavement structure.

The bridge deck pavement structure not only withstands the combined effect of the environmental circumstances (such as temperature, humidity, etc.) and the traffic loads, but also hinders the infiltration of harmful substances such as moisture and oil, which play a key role in the entire bridge structure. Owing to the particularity of the bridge deck pavement structure under the combined effect of external and internal factors, its stress and deformation are more complex than that of highway pavement or airport pavement [9]. For this reason, double-layered modified asphalt pavement structure is often utilized in bridge deck pavement structures. In this study, the upper layer of the pavement was selected as an open gradation friction course (OGFC) with a nominal maximum aggregate size (NMAS) of 13 mm, OGFC-13, and the lower layer of the pavement was asphalt concrete (AC) mixture with the NMAS of 13 mm, AC-13. The type of asphalt binder used in the mixture was a high viscoelastic modified asphalt. The structural system of the bridge deck pavement is shown in Figure 1.

**Figure 2:**
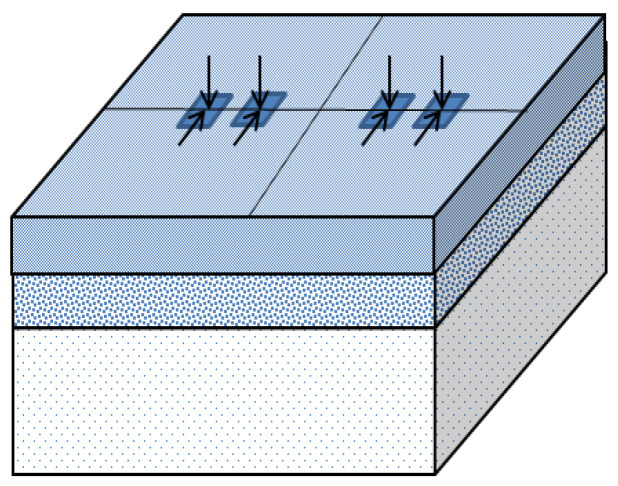
Schematic diagram and load arrangement of the bridge deck pavement.

The model has the dimensions of 6 m × 6 m on the horizontal plane, and the dimension of the vertical plane is equal to the thickness of the structural system. The distribution of the loading area is positioned in the middle of the surface of the model structure, as shown in Figure 2.

**Figure 3:**
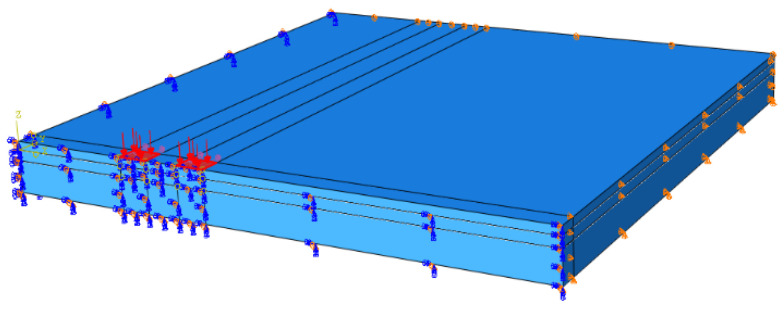
Boundary conditions of 1/4 three-dimensional FE model.

Considering the symmetry of the load and structure, only 1/4 of the model was used for calculation and analysis. The boundary condition was fixed at the bottom of the bridge deck. In addition, no transverse horizontal displacement in the transverse direction of the bridge and no longitudinal horizontal displacement in the longitudinal direction of the bridge were assumed, as shown in Figure 3.

**Figure 4:**
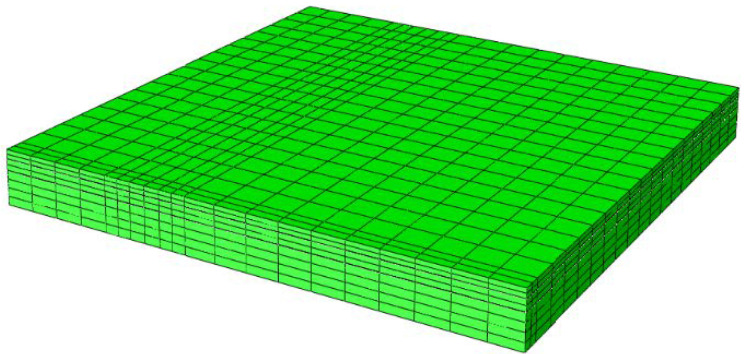
**The** meshing of 1/4 three-dimensional FE model.

Element type and size are imperative for the accuracy of a finite element (FE) analysis. Twenty-node brick element with reduced integration (C3D8R) was adopted for meshing. The finest mesh was used around the loading area with rectangular surface contact, and the meshing size gradually widened as it recedes from the loading area to decrease the computation time (Figure 4). The analysis temperature was set to 25 °C, and other structural and material parameters are displayed in Table 1. It should be noted that all materials were characterized as elastic materials.

#### 2.1.2. Loading Application

**Figure 5:**
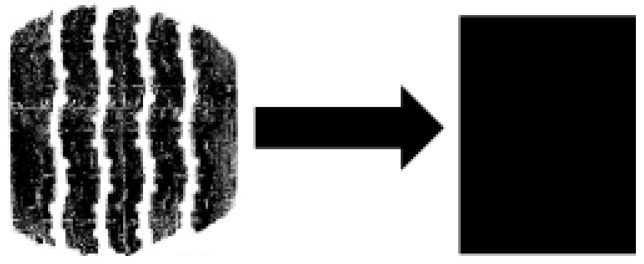
Schematic diagram of tire imprint.

In this study, a moving 100 kN single axle load with dual tire assembly, which is the standard axle load for the structural design of asphalt pavement in China, was applied to the loading area. Moreover, the effects of horizontal and vertical loadings were considered concurrently. The horizontal load is obtained by multiplying the vertical load and the horizontal load coefficient. In this study, the coefficient of horizontal force was set as 0.5. In the mechanistic pavement design method, it is assumed that the approximate shape of the contact area for each tire consists of a rectangle and two semicircles with length L and width 0.6 L [36]. The length can be calculated using Equation (1):

(1) $$L=\sqrt{\frac{A_{c}}{0.5227}}\;$$

where *A_c_* is the contact area calculated by dividing the load on each tire by the tire pressure. In the FE procedure, the contact area is simplified as an equivalent rectangular area with length 0.8712 *L* and width 0.6 *L* (Figure 5). 

**Figure 6:**
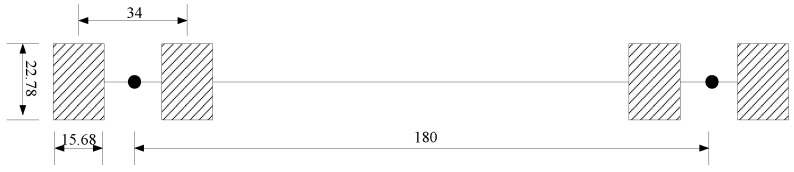
Schematic diagram of load arrangement (unit: cm).

In this study, the axle load is 100 kN and the tire pressure is 0.7 MPa. Therefore, it is calculated that the tire width is 15.68 cm, and the tire length is 22.78 cm. The schematic diagram of load distribution after a simplified calculation is shown in Figure 6.

Nonuniform vertical compression stresses have a single direction along the longitudinal contact length, and a half-sinusoidal pressure is assumed for its distribution, as shown in Equation (2).

(2) $$P\left(t\right)=p\cdot \sin^{2}\left(\frac{\pi t}{T}\right)\;$$

The duration of load depends on the vehicle speed and the tire contact radius as follows:

(3) $$T=\frac{12R}{v}\;$$

where *p* is the maximum contact pressure which is 0.70 MPa; *T* is the duration of load, s; v is the vehicle speed, m·s^−1^; and *R* is the tire contact radius, m, which is 0.1065 m for standard axel load. The driving speed is set as 80 km/h, and the calculated load duration is 0.058 s.

#### 2.1.3. Interfacial Conditions

For the bonding conditions of the adhesive layer (upper interface) and waterproof adhesive layer (lower interface) in bridge deck pavement structure, five different bonding conditions were considered as a function of the friction coefficient (μ). The coefficient is used to characterize different bonding states, which are full slip (μ = 0), full bonding (μ = ∞), and partial bonding (μ = 0.25, 0.5, and 0.75). On this basis, μ_1_ represents the bonding state of the upper interface and μ_2_ represents the bonding state of the lower interface. In this study, when the influence of the upper interlayer bonding conditions on the shear stress at the bottom of the pavement structure is concerned, it was assumed that the lower interlayer is in partial bonding condition (i.e., μ_2_ = 0.75) and vice versa.

## 3. Experimental Program

### 3.1. Materials

**Figure 7:**
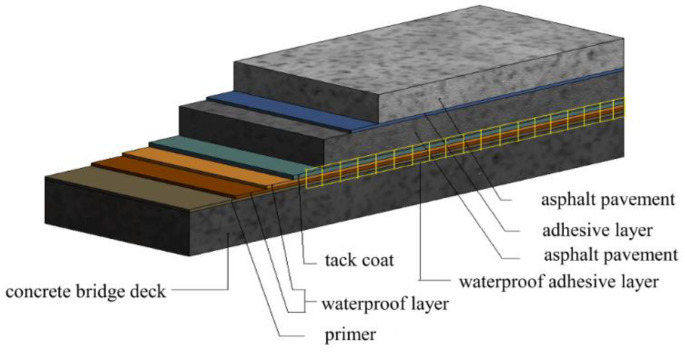
Schematic diagram of bridge deck pavement structure.

To improve the shear resistance between layers of bridge deck pavement and facilitate reasonable material selection, in this study, varying typical bonding materials were selected to determine their shear bond strength through the direct shear test. As shown in Figure 7, three different types of adhesive materials, resin emulsified asphalt, modified emulsified asphalt, and ordinary emulsified asphalt were selected as bonding materials between the layers of the bridge deck pavement structure. OGFC-10 mixture was used as the upper layer of the pavement and AC-13 mixture was employed as the lower layer of the pavement, overlaying the concrete bridge deck.

The aggregate gradation of the mixtures is presented in Table 2. In addition, basalt as coarse aggregate, machine-made sand as fine aggregate, alkaline mineral powder as filler, and a high viscoelastic modified asphalt as asphalt binder were utilized for preparing the asphalt mixtures. The bridge deck was made up of the C40 ordinary cement concrete, and the mix proportion was selected as cement: water: sand: gravel = 347.5:139:543:1396. The test temperature was set as room temperature at 25 °C.

### 3.2. Specimen Preparation

For manufacturing of the double-layered asphalt specimens, first, the lower layer AC-13 mixture was prepared by 32 times single-sided compaction. The compacted specimen was then allowed to cool to room temperature. After cooling, an appropriate amount of bonding material was applied to the surface and then the coated specimen was set aside to cure. Then, the upper layer OGFC-10 loose mixture was formed by 32 times single-sided compaction.

**Figure 8:**
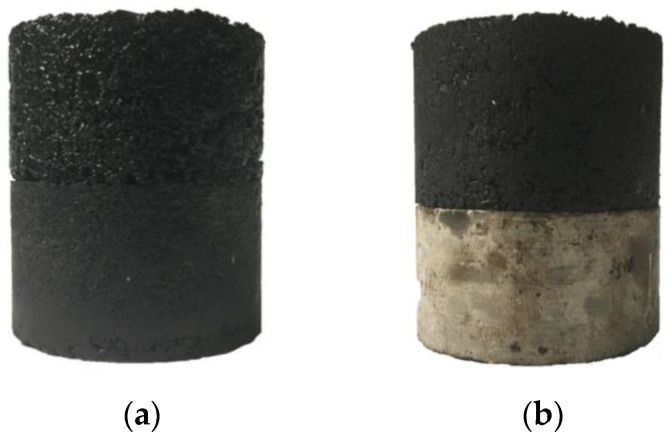
Double-layered specimen: (**a**) double-layered asphalt specimen (system I); (**b**) double-layered composite specimen (system II).

Similarly, for the fabrication of double-layered composite specimens, first, the lower half concrete layer with a thickness of 32 mm was obtained as a core specimen and its surface was sandblasted. After cleaning the surface, an appropriate amount of bonding materials was applied to surface. Subsequently, the coated surface was set aside at room temperature for 2 h to allow the curing procedure completed. Following this, the AC-13 mixture was poured into the second half of the mold and prepared by 32 times single-sided compaction. To distinguish two interlayers from each other, the interlayer between the upper and lower asphalt layers is called the upper interlayer, and the interlayer between the lower asphalt layer and the concrete layer is called the lower interlayer. Images of two types of double-layered specimens are presented in Figure 8.

### 3.3. Test Device

**Figure 9:**
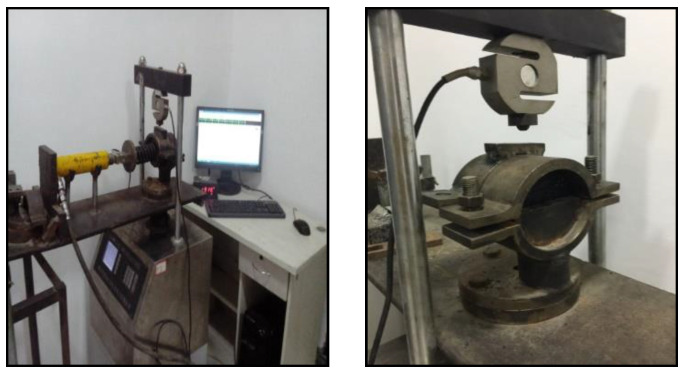
The direct shear test set up.

A supplementary fixture was developed and attached to a Marshall testing machine to conduct the interlayer bonding evaluation of the double-layered specimens, as shown in Figure 9. The direct shear strength of the interface was reported as the test result.

## 4. Results and Discussion

### 4.1. Simulation Results

In this section, the effect of the bonding condition at the upper and lower interfaces on the pavement responses at the bottom of the upper and lower layers will be analyzed and discussed.

#### 4.1.1. Shear Stress in the Upper Layer

(a) Effect of upper interlayer bonding condition

**Figure 10:**
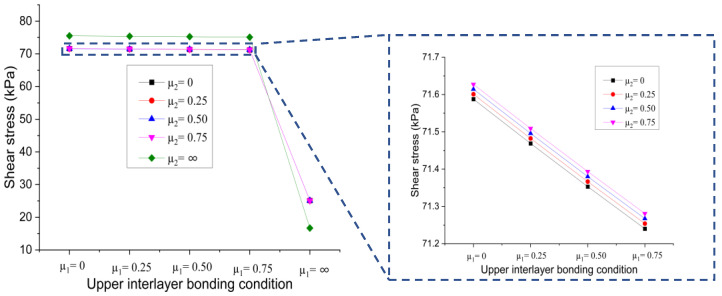
Influence of upper interlayer bonding condition on shear stress at the bottom of upper layer.

Figure 10 shows the change trends of the shear stress at the bottom of the upper layer against the upper interlayer bonding condition when the contact state of the lower interlayer changes from full slip to full bonding. 

It can be seen that the shear stress at the bottom of the upper layer reduced with the increase of the friction coefficient at the upper interface, regardless of the bonding conditions at the lower interface. The results reveal that when the bonding condition of the lower interface is unchanged, the better bonding condition at upper interface causes the lesser shear damage to the interlayer generated because of loading. Therefore, there is a remote possibility of upper interface failure. In addition, when the bonding conditions at upper and lower interfaces are in a full bonding state, the resulting shear stress at the bottom of the upper pavement layer is minimum. When the lower interface is in the full slip and partial bonding states, the curves show a linear downward trend, and the rate of decline is almost the same. With the increase of the friction coefficient of the upper interface to the full bonding state, the resulting shear stress declined to the lowest point, which is about 35% of that of the full slip state. On the other hand, when the lower interface is in the full bonding state, the curve also shows a linear downward trend, but the downward rate is larger. In conclusion, different bonding conditions of the upper and lower interface produce different effects on the shear stress at the bottom of the upper layer.

(b) Effect of lower interlayer bonding condition

**Figure 11:**
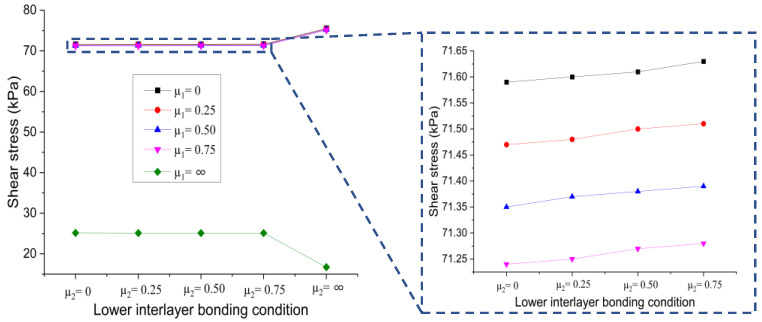
Influence of lower interlayer bonding condition on shear stress at the bottom of upper layer.

Figure 11 illustrates the changing trend of the shear stress at the bottom of the upper layer against the lower interlayer bonding condition when the contact state of the upper interlayer changes from full slip to full bonding.

It is evident that when the lower interlayer is in a full slip and partial bonding state, the resulting shear stress at the bottom of the upper layer gradually increased with increasing the friction coefficient of the lower interlayer. Moreover, when the lower interlayer is in full bonding condition, the shear stress reduced with the increase of the friction coefficient of the lower interlayer. On the other hand, when the upper interlayer is in the state of the full slip or partial bonding, the increase of the friction coefficient of the lower interlayer is not necessarily conducive to the shear resistance of the upper interlayer. Only when the friction coefficient of the upper interlayer increased to a certain extent did the increase of the friction coefficient of the lower interlayer lead to the resistance of the upper interlayer to shear stress.

#### 4.1.2. Shear Stress in the Lower Layer

(a) Effect of upper interlayer bonding condition

**Figure 12:**
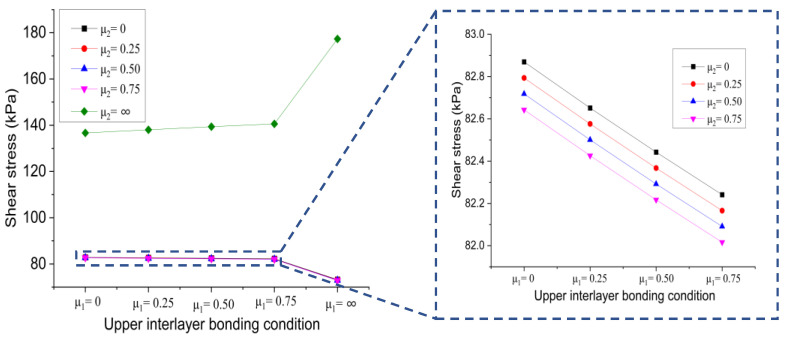
Influence of upper interlayer bonding condition on shear stress at the bottom of lower layer.

Figure 12 displays the changing trend of the shear stress at the bottom of the lower layer versus the upper interlayer bonding condition when the contact state of the lower interlayer is changes from full slip to full bonding.

It can be seen that when the lower interlayer is in the full slip and partial bonding state, the shear stress at the bottom of the lower layer decreased linearly with the increase of the friction coefficient of the upper interlayer, and the decline rate of different curves was almost the same. When the contact state of the upper interlayer reached the full bonding state, the resulting shear stress reached the minimum, which is about 88% of that of the full slip state. When the lower interlayer is in full bonding condition, the shear stress gradually increased with the increase of the friction coefficient of the upper interlayer and reached the maximum value when the upper interlayer is in a full bonding state. In this case, the shear stress is about 1.3 times that of the full slip state. It can be inferred that varying bonding conditions at the upper interlayer will have different effects on the resulting shear stress at the bottom of the lower layer. In all cases, increasing the friction coefficient of the upper interlayer led to a decrease in shear stress at the bottom of the lower layer. However, when the bonding condition at the lower interlayer is in a full bonding state, increasing the friction coefficient at the upper interface will increase the shear stress at the bottom of the layer, which is not beneficial to the interlayer performance.

(b) Effect of lower interlayer bonding condition

**Figure 13:**
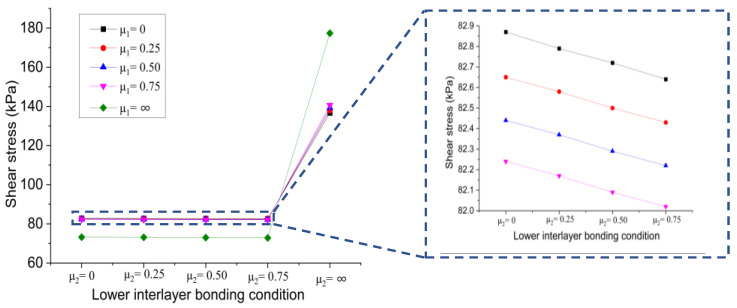
Influence of lower interlayer bonding condition on shear stress at the bottom of lower layer.

Figure 13 shows the changing trend of the shear stress at the bottom of the lower layer versus the lower interlayer bonding condition when the contact state of the upper interlayer is changes from full slip to full bonding. 

It is evident that the shear stress at the bottom of the lower layer decreased first and then increased with the increase of the friction coefficient of the lower interlayer, regardless of the contact state of the upper interlayer. Especially, when the bonding condition of the lower interlayer is full bonding, the resulting shear stress at the bottom of the lower layer was significantly increased, which is unfavorable to the overall performance of the bridge deck pavement structure.

To sum up, it can be inferred that no matter what contact state the lower interlayer is in, the shear stress at the bottom of the upper pavement layer gradually decreased with the increase of the friction coefficient of the upper interlayer, and the lower interlayer in different contact states had an impact on the change rate of the curve, but the impact was very small. In addition, only when the friction coefficient of the upper interlayer was large was the increase of the friction coefficient of the lower interlayer conducive to reducing the shear stress at the bottom of the upper pavement layer. Finally, different bonding conditions of the upper interlayer will have significantly different effects on the shear stress and at the bottom of the lower pavement layer. When the lower interlayer bonding condition is in full slip or partially bonding condition, then increasing the friction coefficient of the upper interlayer is conducive to reducing the shear stress at the bottom of the lower pavement layer. It was reported that better interface bonding condition could enhance the resistance to bottom-up fatigue cracking significantly for thin (40 mm-thick) deck pavements [32].

### 4.2. Experimental Results

Table 3 displays the interlayer bond strength results for each system and bonding material type under three temperature conditions. For each test condition, three replicates were prepared, and the average value was reported as test results. The effects of adhesive material and structure type on interlayer bonding performance were discussed in the following section.

#### 4.2.1. Effect of Bonding Material

**Figure 14:**
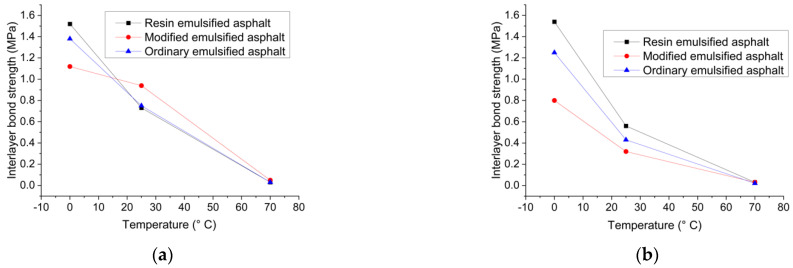
Interlayer shear strength of three adhesive materials in two structures: (**a**) Structure I; (**b**) Structure II.

Figure 14 illustrates the variation curves of interlayer shear strength against temperature for three different adhesive materials in structure I and Structure II respectively. 

It can be seen that the interlayer shear bond strength formed by the three adhesive layer materials decreased significantly with the increase of temperature in both bridge deck pavement structures, but there are significant differences in the changing trend of the interlayer bond strength with temperature. These results are in agreement with the findings of previous studies [37]. This indicates that the temperature sensitivity of the interlayer bonding materials exists in all kinds of bridge deck structures, and there are significant differences in the sensitivity of different interlayer treatments. Currently, most of the distresses caused by the interlayer problems are related to the excessive temperature sensitivity of the interlayer bonding materials. The excessive sensitivity leads to the sharp decrease of the direct shear strength of the interlayer, which cannot provide sufficient resistance to shear stress, and finally leads to the occurrence of corresponding distresses. For example, the direct shear strength of the interlayer formed by resin emulsified asphalt and ordinary emulsified asphalt materials in Figure 14a is 1.52 MPa and 1.38 MPa respectively when the temperature is 0 °C. It is evident that there are limited shear distresses at the interface at this stage, indicating that the interlayer has sufficient resistance to shear damage. However, when the temperature increased to 25 °C, the corresponding interlayer bonding strength dropped to 0.73 MPa and 0.75 MPa, respectively. In this condition, the interlayer bonding cannot fully resist resulting in shear damage. When the temperature continues to rise to 70 °C, the interlayer shear strength decreased to 0.03 MPa. At this stage, the bridge deck pavement structure experiences a high possibility of interlayer shear failure. When the severity of shear bonding failure is large, the ability of the interlayer to resist shear damage is seriously insufficient at this stage. To sum up, when an adhesive material is selected for interlayer treatment, interlayer bonding performance and the influence of temperature dependency of interlayer treatment should be considered concurrently.

It can also be seen from Figure 14 that, independent of structure type, when the temperature is normal or low, the interlayer shear strength formed by different adhesive materials is usually different at the same temperature. However, when the temperature increased to high elevation, all the interlayer shear strengths gradually tend to be consistent and reached close to 0 MPa. The reason is that all adhesive materials are temperature dependent. When the temperature is low, the adhesive material itself is stiff and has certain rigidity and strength. In this condition, the interlayer bond strength between the upper and lower layers of the bridge deck pavement has a certain resistance to damage. When the temperature gradually rises to the high temperature, the adhesive material is gradually softened into a plastic state or even a flowing state. In this case, the adhesive material does not play a bonding role, and the friction between the upper and lower layers of the interlayer is greatly weakened. Therefore, the interlayer bonding almost loses the ability to resist shear damage at high temperatures, which also explains why the bridge deck pavement structure is prone to delamination, rutting, and other distresses in high-temperature areas in summer. When selecting a reasonable adhesive material, not only the adhesive ability of the material but also the stability of the interfacial bonding performance is equally important. Zhou et al. [38] also demonstrated that the resin-type waterproof adhesive layer materials have good impermeability, bonding performance, temperature stability, and construction damage resistance in bridge deck pavement.

#### 4.2.2. Effect of Structure Type

**Figure 15:**
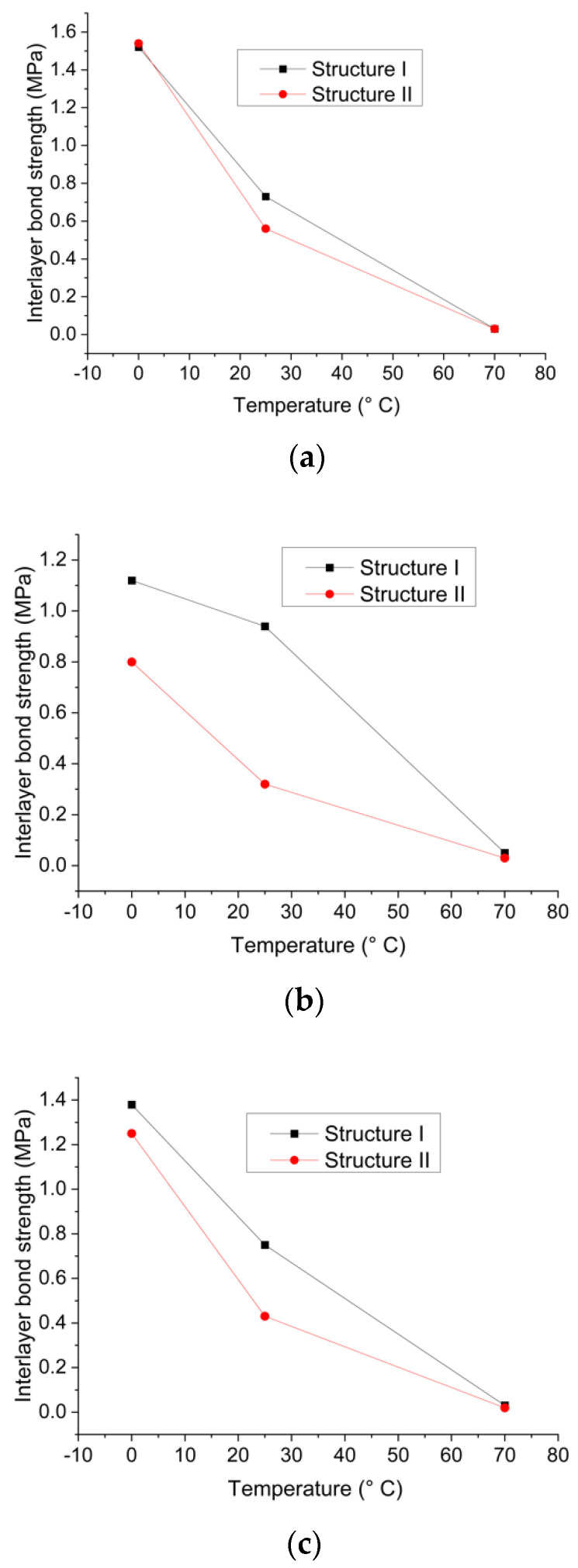
Interlayer shear strength of the same adhesive material in each structure: (**a**) Resin emulsified asphalt; (**b**) Modified emulsified asphalt; (**c**) Ordinary emulsified asphalt.

Figure 15 shows the changing trend of the interlayer shear strength formed by three adhesive materials against the temperature in structures I and II.

It can be seen that the interlayer shear strength of the two bridge deck pavement structures, regardless of the type of adhesive material, decreased significantly with the increase in temperature, but the changing trend of the same material in different structures was different. For example, in Figure 15b, although the two curves exhibited an obvious downward trend with the increase in temperature, structure I was less sensitive at low temperature, and the intensity of the decline rate was moderate. On the other hand, the sensitivity of interlayer bond strength is greater at high temperatures, and the decline rate is sharp. However, the interlayer bond strength of structure II is more prone to low temperature, and the bonding strength decreases significantly. When the temperature is higher, the temperature sensitivity is smaller, and the interlayer bonding strength decreases moderately. The results demonstrate that the degree of temperature dependency of the interlayer bond strength formed by the same adhesive material in different structures is not comparable, leading to the obvious variation trend of the interlayer bond shear properties. Consequently, when a bonding material exhibits a good bond performance in a certain pavement structure, it is not necessarily suitable for other pavement structures. In conclusion, when the bonding performance of certain adhesive material in different pavement structures is compared, the evaluation of performance under various working conditions should be considered rather than comparing the performance, for example, at a certain temperature.

It can also be found from Figure 1 that no matter what kind of adhesive material is utilized, the direct shear performance of the interlayer in two structures is not identical at the same temperature, and the degree of difference is not consistent at different temperatures. Moreover, the results show that the same adhesive material will exhibit different bonding abilities in different structures, resulting in significant differences in the interlayer shear properties. Therefore, for the same adhesive material, the type of pavement structure is also one of the important factors affecting the shear resistance of the interlayer bonding interface in bridge deck pavement. For instance, in Figure 15b, when the temperature is 0 °C, the interlayer shear strength of structure I is 1.12 Mpa while that of structure II is 0.8 Mpa. In this case, the interlayer shear strength of structure II is only 71% of structure I at 0 °C. When the temperature is 25 °C, the shear strength of structure I is 0.94 Mpa and that of structure II is 0.32 Mpa, which is only 34% of structure I. This indicates that the modified emulsified asphalt is suitable for structure I but not for structure II. Therefore, a good interlayer bonding performance is not the superposition of excellent pavement structure and good bonding material, but the optimal combination of a pavement structure and a bonding material. In practical engineering, it is necessary to select the appropriate bonding material according to the specific pavement structure, not necessarily the one with the strongest bonding strength.

In conclusion, there are differences in the interlayer shear performance whether different interlayer treatments are applied under the same pavement structure, or the same interlayer treatment is applied under different pavement structures. Hence, it is imperative to ensure that the interlayer bonding has sufficient resistance against shear failure.

#### 4.2.3. Analysis of Variance

Based on the above results it can be found that structure type, type of adhesive material, and temperature affect interlayer bond strength. However, the significance level of each factor is unknown. For this reason, the statistical analysis of variance (ANOVA) was used to determine the significance of each factor for interlayer bond strength. The analysis was conducted according to data obtained from the test program in this study. ANOVA sets up a collection of statistical procedures to quantitatively specify whether certain parameters and their combinations affect particular responses, which is interlayer bond strength in this study.

The *p*-value is the minimum level of significance at which the factor is considered significant in influencing the response. In this study, the *p*-value of 0.05, i.e., a confidence level of 95% was considered. Table 4 presents the ANOVA results for interlayer bond strength.

As shown in Table 4, *p*-values of all factors are all less than the significant level of 0.05. In other words, all studied factors have a significant effect on the interlayer bond strength. The *F*-value of each factor signifies that the significant effect of each factor on interlayer bond strength in the order of importance ranks as temperature, type of system, and type of adhesive material type.

## 5. Conclusions

The following conclusions can be drawn from the numerical and experimental results of this study:

(1) Regardless of the bonding condition of the lower interlayer, the shear stress at the bottom of the upper layer gradually decreased with the increase of the friction coefficient of the upper layer, and the bonding condition of the lower interlayer had an insignificant effect on the produced shear stress. Only when the friction coefficient of the upper interlayer was large was the increase of the friction coefficient of the lower interlayer conducive to reducing the shear stress at the bottom of the upper layer.
(2) Different bonding conditions of the upper interlayer produced different effects on the shear stress at the bottom of the lower layer. Only when the lower interlayer was in a full slip or partial bonding state was increasing the friction coefficient of the upper interlayer conducive to reducing the shear stress at the bottom of the lower layer.
(3) Regardless of the bonding condition of the upper interlayer, with the increase of the friction coefficient of the lower interlayer, the shear stress at the bottom of the lower layer reduced first and then increased.
(4) When a bonding material is selected for interlayer treatment, interlayer bonding performance and the influence of temperature dependency of interlayer treatment should be considered concurrently. The bridge deck pavement structure is prone to delamination, rutting, and other distresses in high-temperature areas in summer. Therefore, when a bonding material is selected for interlayer treatment, not only the adhesive ability of the material but also the stability of the interfacial bonding performance is equally important.
(5) When the bonding performance of certain adhesive material in different pavement structures is compared, the evaluation of performance under various working conditions should be considered rather than comparing the performance for example at a certain temperature. In practical engineering, it is imperative to select the appropriate bonding material according to the specific pavement structure, not necessarily the one with the strongest bonding strength.
(6) Results of statistical analysis revealed that all factors, including structure type, type of adhesive material, and temperature statistically have a significant effect on interlayer bonding strength.

## Figures and Tables

**Table 1:** Properties of bridge deck pavement layers.

Material	Thickness (cm)	Elastic Modulus (MPa)	Density (g·cm^−3^)	Poisson Ratio
Upper layer	4	2000	2.20	0.3
Lower layer	5	3000	2.20	0.3
Concrete	15	31,000	2.40	0.15

**Table 2:** Design parameters of OGFC-10 and AC-13.

Passing Percentage (%)										Mineral Powder	Fiber (%) *	Asphalt Binder (%) *
Sieve Size (mm)	13.2	9.5	4.75	2.36	1.18	0.6	0.3	0.15	0.075			
OGFC-10	0	3	5	53	7	7	7	4	4	10	0.3	6.2
AC-13	5	33	35	7	1	3	3	1	2	10	0.3	5.8

* Note: The amount of fiber and asphalt is the percentage of the total amount of mixture.

**Table 3:** Interface bond strength, (MPa).

System Type	Adhesive Material	Temperature (°C)		
		0	25	70
Type I	Resin emulsified asphalt	1.52	0.73	0.03
	Modified emulsified asphalt	1.12	0.94	0.05
	Ordinary emulsified asphalt	1.38	0.75	0.03
Type II	Resin emulsified asphalt	1.54	0.56	0.03
	Modified emulsified asphalt	0.8	0.32	0.03
	Ordinary emulsified asphalt	1.25	0.43	0.02

**Table 4:** ANOVA analysis for interlayer bond strength.

Factor	DF ^a^	SS ^b^	MS ^c^	*F*-Value ^d^	*p*-Value ^e^
System type	1	0.399	0.399	93.042	≤0.001
Adhesive material	2	0.353	0.176	41.123	≤0.001
Temperature	2	13.860	6.930	1616.517	≤0.001
Error	36	0.154	0.004		
Total	53	15.932			

^a^ Degrees of freedom. ^b^ Sum of squares. ^c^ Mean square, which is the SS divided by DF. ^d^ Ratio of mean squares. It is used to determine the *p*-value. ^e^ Factor is significant (*p*-value < 0.05).

## Data Availability

Not applicable.
